# Supplementary material for: Light-induced charge generation in polymeric nanoparticles restores vision in advanced-stage retinitis pigmentosa rats
Source: Nat Commun. 2022 Jun 27;13:3677. doi: 10.1038/s41467-022-31368-3 (PMC9237035; doi:10.1038/s41467-022-31368-3)
Supplement: Supplementary file 1 — Supplementary Information [file 41467_2022_31368_MOESM1_ESM.pdf]

LIGHT-INDUCED CHARGE GENERATION IN POLYMERIC NANOPARTICLES RESTORES VISION IN ADVANCED-STAGE RETINITIS PIGMENTOSA RATS

S. Francia[1,3]*, D. Shmal[1,2]*, S. Di Marco[1,3]*, G. Chiaravalli[4], J.F. Maya-Vetencourt[1,5], G. Mantero[1,2], C. Michetti[1,2], S. Cupini[1,2], G. Manfredi [4,6], M.L. DiFrancesco[1,3], A. Rocchi[1], S. Perotto[4], M. Attanasio[7], R. Sacco[8], S. Bisti[1], M. Mete[7], G. Pertile[7], G. Lanzani[4,9], E. Colombo[1,3]*, F. Benfenati[1,3]*

[1]Center for Synaptic Neuroscience and Technology, Istituto Italiano di Tecnologia, Genova, Italy; [2]Department of Experimental Medicine, University of Genova, Italy; [3]IRCCS Ospedale Policlinico San Martino, Genova, Italy; [4] Center for Nanoscience and Technology, Istituto Italiano di Tecnologia, Milano, Italy; [5]Department of Biology, University of Pisa, Pisa, Italy; [6]Novavido s.r.l, Bologna, Italy; [7]Department of Ophthalmology, IRCCS Sacrocuore Don Calabria Hospital, Negrar, Verona, Italy; [8]Department of Mathematics, Politecnico di Milano, Milano, Italy; [8]Department of Physics, Politecnico di Milano, Milan, Italy.

* Equal contribution

Correspondence should be addressed to F.B. (Email: fabio.benfenati@iit.it) and G.L. (Email: guglielmo.lanzani@iit.it)

SUPPLEMENTARY INFORMATION

Supplementary Text

Nanoparticle Interaction with the Biological Environment

Photostimulation by P3HT-Nanoparticles (NPs) was proposed to consist in the photo-induced electrical polarization and subsequent capacitive coupling of the NPs in tight "*gigaseal*" contact with the neuronal membrane. First, we provide here the general picture on which we base our modeling. Following light absorption in the nanoparticle, the larger fraction of singlet states (often referred to as “excitons”) decays quickly to ground state (1 ns lifetime). Polaron-pairs, generated in smaller number, follow a similar fate due to the quick geminate recombination. Eventually there is however an additional, small fraction of states that survive to the ms time scale and can thus build up under prolonged illumination (> ms). These are byproducts of a number of processes, which we do not consider here in detail. We just consider, based on experiments, that long-lived separated charge pairs are formed in the crystalline domains of the NP, as expected for regio-regular P3HT (Jiang et al., 2002). Note that there are amorphous regions as well in the NPs, yet those do not play a role in the build-up of a long-lived carriers' population, as ultrafast energy transfer takes place from the amorphous to the crystalline phase (Bargigia et al., 2018). The photoconversion of incident light into charge carriers and their successive transport throughout the NP bulk is taken into account by performing suitable modeling approximations, as described in (Chiaravalli et al., 2021).

The initial Lambert-Beer concentration profile in space drives carrier diffusion, leading to an excess of negative charge at the illuminated surface and to an excess of positive charge at the opposite side because of the well-known asymmetry in carrier mobility (Chiaravalli et al., 2021): holes quickly spread out homogeneously in the film, while electrons (negative polarons) are essentially fixed. The photogenerated negative carriers that reach the interfaces are assumed to reduce molecular oxygen dissolved in the retinal environment and exit the nanoparticle with a dynamic described by the Marcus-Gerischer model.

Furthermore, we speculate that the cleft between the NP and the cell membrane is a dielectric medium with high resistivity made by adhesion proteins that establish a soft interface, diffused over tens of nanometers. Hydrated oxygen in this dielectric layer induces electron acceptor states that stabilize the negative charge giving rise to a sizable electrical potential which, under certain conditions, spreads across several hundreds of nanometers in the cleft. All the above-reported mechanisms have been formalized and reproduced with the use of mathematical

modeling as described in detail in the next sections. We note here that the model does not depict the complex interface with the neuron, that will in general be an active player in the coupling. The model regards the effect of light on the NP embedded in a non-homogeneous space. The region of the cleft has quite different electrical screening and conductivity properties than the extracellular medium. The simulation provides the spatial range of the electrical potential generated at the NP surface. This will affect the membrane of second-order retinal neurons.

Mathematical Model

To mathematically describe the physical mechanisms occurring at the interface between the NP and the biological environment, we solve a stationary non-linear system of partial differential equations accounting for the description of the semiconductor nanoparticle coupled with the surrounding biological environment. The nanoparticle is modeled with the use of the classical Drift Diffusion equations, coherently to what done in (Chiaravalli et al., 2021), whereas the electro-diffusion of ions is modeled with the Poisson-Nernst-Planck (PNP) model (Sacco et al., 2019). The DD and PNP equations are coupled through the use of appropriate conditions at the interface between the NP and the extracellular medium and cleft, respectively.

We have iteratively solved the nonlinear system of partial differential equations by means of Gummel's decoupled algorithm (Jerome, 1996; Gummel, 1964) and the resulting linearized equations are discretized with piecewise linear continuous finite elements. To ensure strict positivity of the computed carrier and ion number densities, the Scharfetter-Gummel stabilization is introduced in the discretized continuity equations (Scharfetter et al., 1969, Sacco et al., 2019). The computational algorithm is in-house coded and has been implemented using the MatLab environment. The values of model parameters are the same as in (Chiaravalli et al., 2021) and/or reported in **Supplementary Table 1.**

The domain

The device object of our analysis and simulation is schematically represented in **Supplementary Fig. 5**. We denote by Ω the one- dimensional open interval (0,W), comprising three subdomains, $\Omega = \{\Omega_1, \Omega_2, \Omega_3\}$. The interface between the P3HT-NP and the cleft is located at x = R_1 whereas the interface between the P3HT-NP and the extracellular medium is located at x = R_2. For each domain Ω_i, i=1,2,3, we denote by $\partial\Omega_i$ the boundary of Ω_i and by ν_i the outward unit normal vector on $\partial\Omega_i$. Letting e_x the unit normal vector directed as the

positive x-axis, we see that $\nu_1 = -e_x$ at x = 0 and $\nu_1 = +e_x$ at x = R_1, $\nu_2 = -e_x$ at x = R_1 and $\nu_2 = +e_x$ at x = R_2, $\nu_3 = -e_x$ at x=R_2 and $\nu_3 = +e_x$ at x = W. In the remainder of this text, we assume that photogeneration mechanisms, electronic and ionic transport and electric effects happen solely along the x direction and do not depend on the other spatial coordinates y and z. The nanoparticle diameter is assumed equal to 300 nm, R_1 is placed at 1000 nm and W at 2300 nm. Accordingly, we enforce the boundary conditions for the dependent variables at x = 0 and x = W, far enough from the relevant interfaces so that they cannot affect system behavior too strongly.

Model Equations

In Ω_1 and Ω_3, namely in the cleft and in the extracellular medium, we solve the continuity equations for three ionic species i = Na^+, Cl^- and O_2^-.

In the case where i = Na^+, Cl^-, we solve a continuity equation with a null right-hand side:

$$\nabla_x \cdot J_i = 0 \tag{1a}$$

$$J_i = q\mu_i \frac{z_i}{|z_i|} N_{av}\, c_i\, E - qN_{av}D_i \nabla_x c_i \tag{1b}$$

where q is the elementary charge [C], N_{av} is the Avogadro constant [mol^{-1}] and for i = Na^+, Cl^- J_i is the current density [C m^{-2} s^{-1}], z_i is the chemical valence, μ_i is the electric mobility [V m^{-2} s^{-1}], D_i is the diffusivity [m^2 s^{-1}] and c_i the molar density [mol m^{-3}]. Diffusivity and electric mobility are related by the Einstein-Smoluchowski equation.

The O_2^- ions are supposed to have a lifetime τ_{ox} [s], which effectively describes the capacity of O_2^- ions to react with species in solution. For i = O_2^-, we have:

$$-\nabla_x \cdot J_{O_2^-} = -\frac{c_{O_2^-}}{\tau_{ox}} \tag{2a}$$

$$J_{O_2^-} = +q\mu_{O_2^-} N_{av}\, c_{O_2^-}\, E + qN_{av}D_{O_2^-} \nabla_x c_{O_2^-} \tag{2b}$$

In the nanoparticle we solve the continuity equations for the two photogenerated carriers, namely, holes, whose number density is denoted by *p* [m^{-3}]:

$$\nabla_x \cdot J_p = q\left(\eta_{diss}\, G_{light}(x) - \frac{pn - n_i^2}{\tau_n (p + n_i) + \tau_p (n + n_i)}\right) \tag{3a}$$

$$J_p = +q\mu_p pE - qD_p \nabla_x p \tag{3b}$$

and electrons, whose number density is denoted by *n* [m^{-3}]:

$$-\nabla_x \cdot J_n = q\left(\eta_{diss}\, G_{light}(x) - \frac{pn - n_i^2}{\tau_n\,(p+n_i) + \tau_p(n+n_i)}\right) \tag{4a}$$

$$J_n = +q\mu_n n\mathrm{E} + qD_n\,\nabla_\mathrm{x}\mathrm{n} \tag{4b}$$

As described in (Chiaravalli et al., 2021), η_{diss} is an effective parameter describing the photoconversion efficiency of photons into free carriers, whereas G_{light} is a given function of position describing the photogeneration rate inside the bulk of the NP [m^{-3} s^{-1}]. G_{light} assumes a different mathematical expression depending on the illumination side: when light comes from the cleft side $G_{light} = I_0\alpha\, exp(-\alpha x)$ and $G_{light} = I_0\alpha\, exp\big(-\alpha(1-x)\big)$ when light comes from the extracellular side. I_0 is a light intensity [photons m^{-2} s^{-1}] and α is the absorption coefficient [m^{-1}]. The second term on the right-hand side of Eq. (3a) and Eq. (4a) represents the net recombination rate in the bulk of the NP according to the Shockley-Read-Hall theory, $\tau_n$and τ_p being the carrier lifetimes [s] and n_i the intrinsic concentration of carriers inside the nanoparticle [m^{-3}].

The Poisson equation is solved in Ω, with a piecewise definition of the right-hand side across the various subdomains, as reported below. We have that:

$$\nabla_x \cdot D = qN_{av}\left(\sum^{\mathrm{i}} z_i\, c_i\right) \qquad \text{for } \mathrm{x} \in \Omega_{1,}\Omega_{3,} \tag{5a}$$

$$\nabla_x \cdot \mathrm{D} = q(\mathrm{p}-\mathrm{n}) \qquad \text{for } \mathrm{x} \in \Omega_2 \tag{5b}$$

In equations (5), the electric displacement is defined as $D = \varepsilon E$ where E is the electric field related to the electric potential φ through the equation: $E = -\frac{\partial\varphi}{\partial x}$.

The Boundary Conditions

In the case of the continuity equations (1) with i=Na^+, Cl^- we set:

At x = 0	$\mathrm{c_i} = 0.1\,\mathrm{mM}$	(6a)
At x = W	$c_i = 100\, mM$	(6b)
At x = R_1, R_2	$J_i \cdot \nu_i = 0$	(6c)

Conditions (6a) and (6b) amount to assuming the ion molar density to be given at the cleft and extracellular side endpoints, respectively, whereas condition (6c) amounts to assuming that ions cannot flow from the solution into the nanoparticle.

In the case of the continuity equation (2) for i = O_2^-, we set:

At x = 0 $$-J_{O_2^-} \cdot \nu_1 = 0 \quad (7a)$$

At x = W $$-J_{O_2^-} \cdot \nu_3 = 0 \quad (7b)$$

At x = R$_1$ $$-J_{O_2^-} \cdot \nu_1 = -\mathrm{J}_{\mathrm{MG}}|_{\mathrm{x=R_1}} + \mathrm{J}_{\mathrm{REC}}|_{\mathrm{x=R_1}} \quad (7c)$$

At x = R$_2$ $$-J_{O_2^-} \cdot \nu_3 = -J_{MG}|_{x=R_2} + J_{REC}|_{x=R_2} \quad (7d)$$

Having introduced the current densities J_{MG} and J_{REC} defined as:

$$\mathrm{J}_{\mathrm{MG}} = \mathrm{qn_{int}} \frac{k_t \, n_{ox} \, \sigma}{(\pi \, K_B \, T \, \lambda)^{1/2}} \exp\left(-\frac{(E_L - E_F^{ox} - \lambda)^2}{4\lambda \, K_B \, T} \right) \quad (8a)$$

$$\mathrm{J}_{\mathrm{REC}} = q k_p c_{O_2^-,int} N_{av} p_{int} \quad (8b)$$

All the parameters in (8) will be introduced with the boundary conditions for photogenerated carriers.

Conditions (7a) and (7b) amount to assuming that O_2^- ions cannot flow out of the solution, whereas conditions (7c) and (7d) describe the coupling mechanism occurring at the interfaces x=R$_1$, R$_2$ between the photogenerated carriers and the reduced oxygen. When an electron exits the bulk of P3HT, it gets transformed through a redox reaction into a O_2^- molecule, which mainly accumulates at the surface or diffuses inside the environment. The O_2^- molecules that remain near the surface of the nanoparticle also provide centers for recombination of holes.

In the case of the continuity equation (3) for the photogenerated holes, we set:

At x = R$_1$, R$_2$ $$J_p \cdot \nu_2 = \mathrm{J}_{\mathrm{REC}} = q k_p c_{O_2^-,int} N_{av} p_{int} \quad (9)$$

Condition (9) amounts to assuming that holes recombine with the reduced oxygen accumulating at the interface after illumination, where k_p is the hole surface recombination probability [m^4 s^{-1}], $c_{O_2^-,\mathrm{int}}$ is the molar density of O_2^- at x=R$_1$, R$_2$ computed from Eq. (2) and p_{int} is the hole number density at x = R$_1$, R$_2$.

In the case of the continuity equation (4) for the photogenerated electrons, we assume a net current of electrons exiting the bulk of P3HT due to a reaction of reduction of oxygen

$$O_2 + e^- \rightarrow O_2^-$$

This effect is mathematically taken into account in the model by the Marcus-Gerischer theory and applied as boundary condition to Eq. (4):

At x = R$_1$, R$_2$	$-J_n \cdot \nu_2 = \mathrm{J_{MG}} = \mathrm{q n_{int}} \frac{k_t\, n_{ox}\, \sigma}{(\pi\, K_B\, T\, \lambda)^{1/2}} \exp\left(-\frac{(E_L - E_F^{ox} - \lambda)^2}{4\lambda\, K_B\, T} \right)$	(10)

k_t being the tunneling coefficient for electrons in the P3HT [m^4 s^{-1}], $n_{ox}$the concentration [m^{-3}] of molecular oxygen at the interface with the external environment, σ the P3HT disorder parameter [eV], K_B the Boltzmann constant [J K^{-1}], T the temperature of the environment [K] and λ the width of the Gaussian distribution of molecular oxygen states [eV], E_L the energy of the lowest unoccupied molecular orbital (LUMO) of P3HT, E_F^{ox} the energy corresponding to the potential of the oxygen reduction reaction, and n_{int} the electron number density at x = R$_1$, R$_2$ [m^{-3}]. Notice that at each iteration of the Gummel solution map, the value of E_F^{ox} is updated with the following expression:

$$E_F^{ox} = E^0_{O_2/O_2^-} + \frac{RT}{F} ln \frac{c_{O_2}}{c_{O_2^-,int}}$$

where $E^0_{O_2/O_2^-}$ is the standard redox potential for the redox couple assuming a constant atmospheric pressure and temperature, equal to $E^0_{O_2/O_2^-} = -0.33\, V\, vs\, SHE$. The molar concentration of molecular oxygen c_{O_2} is assumed as a constant of the system whether $c_{O_2^-,int}$ is the output at x = R$_1$, R$_2$ of Eq. (2).

In the case of the Poisson equation (5), we set:

At x = 0, W	$\varphi = 0$	(11a)
At x = R$_1$	$D_1 \cdot \nu_1 = c_\Gamma\ (\varphi_1\|_{x=R_1} - \varphi_2\|_{x=R_1})$	(11b)
At x = R$_1$	$D_2 \cdot \nu_2 = c_\Gamma\ (\varphi_2\|_{x=R_1} - \varphi_1\|_{x=R_1})$	(11c)
At x = R$_2$	$D_2 \cdot \nu_2 = c_\Gamma\ (\varphi_2\|_{x=R_2} - \varphi_1\|_{x=R_2})$	(11d)
At x = R$_2$	$D_3 \cdot \nu_3 = c_\Gamma\ (\varphi_3\|_{x=R_2} - \varphi_2\|_{x=R_2})$	(11e)

where, for any continuous function ψ, we indicate by ψ_i the restriction of ψ to Ω_i and $\psi|_x$ the value of ψ at x. Condition (11a) amounts to assuming that the electric potential is set equal to zero at the domain endpoints. Condition (11b) amounts to assuming that the solid-liquid interface behaves like a capacitance, c_Γ = 7.45 F m^{-2} (Tullii et al., 2017) representing the specific capacitance associated with the interface thickness between NP and surrounding electrolyte environment. A similar interpretation applies to conditions (11c), (11d) and (11e).

Simulation Results

To study the effect of the environment on the photoactivation of a P3HT-NP, we have performed several simulations utilizing the model described in the previous sections. All the

reported results (unless otherwise specified) have been obtained considering a light intensity impinging onto the nanoparticle of 10 mW cm^{-2}, a value consistent with the radiation reaching the retina during the pupil reflex experiment, and with a molar density of molecular oxygen of 0.5 mol m^{-3}, characteristic of the retinal environment (Verticchio Vercellin C.A. et al., 2021).

Supplementary Fig. 6 shows hole and electron number densities in the nanoparticle when light impinges from the cleft side. Electrons have a negligible mobility and remain fixed to their position, whereas holes redistribute across the bulk thanks to diffusive gradients: this gives rise to an interface where electrons are available for the reduction reaction in a higher quantity (Chiaravalli G. et al., 2021).

Supplementary Fig. 7 shows the electric potential profile in the three computational subdomains. It is visible that the potential is linear inside the nanoparticle. In the electrolyte environment, instead, it shows two asymmetric behaviors. In the cleft we observe a polarization of about -15 mV in proximity of the interface with the nanoparticle, which decreases reaching a null polarization at about 600 nm away from the interface. This suggests a possible interaction with cells: the cleft is indeed estimated to be about 20 nm, and anyway < 100 nm. In the extracellular side, instead, we observe that the electric potential is three orders of magnitude smaller, due to the efficient screening effect exerted by the conductive extracellular medium.

Supplementary Fig. 8 shows the superoxide molar concentration in the two regions next to the nanoparticle. Coherently with what observed for the potential, considering a standard value for the diffusion coefficient (m^2 s^{-1}), we see that the molar concentration is higher at the interface between cleft and nanoparticle and, instead, reduced at the extracellular side. Notice that, in this condition, at the cleft side, a fraction of 1/5 of the molecular oxygen present at the interface is reduced into O_2^-. The asymmetric production of reduced oxygen is a consequence of the Lambertian profile of generated carriers inside the nanoparticle.

The cleft region is supposed to be filled with proteins and its mathematical characterization is nontrivial: for this reason, we study the behavior of the molar concentration of reduced oxygen in the cleft as a function of the diffusion coefficient $D_{O_2^-}$. We notice that if the diffusion is hampered by the proteinic nature of the cleft, the molar concentration of O_2^- accumulating at the interface with the nanoparticle would be more than doubled, as visible in **Supplementary Fig. 8** (left panel). The increased molar concentration is also reflected by the increase of the electric potential at the interface with the nanoparticle, as visible in **Supplementary Fig. 9a**. By reducing the diffusion coefficient by one order of magnitude, we observe that the potential at the interface almost doubles from about -18 mV to about -32 mV, reaching a value of -53 mV when decreasing the diffusion coefficient by two orders of magnitude.

Supplementary Fig. 9b shows the electric potential in the cleft as a function of the concentration of molecular oxygen. We observe that when no oxygen is available for the reduction, the electric potential at the interface is totally quenched. When, instead, the concentration of molecular O_2 reaches a value typical of the retinal environment, we are able to observe a polarization, which is different from zero up to several hundreds of nanometers.
Supplementary Fig. 9c shows the electric potential in the cleft as a function of the concentration of the screening ions Na^+ and Cl^- inside the cleft. Indeed, in the model we are assuming a highly resistive behavior of the cleft region, which is a crucial condition in order to represent a capacitive interaction between the nanoparticle and the cells surrounding it. The presence of a large number of ions in the solution indeed provides a screening effect of the accumulating O_2^-, thus hampering the polarization of the cleft.
Supplementary Fig. 10 shows the importance of the illumination side in giving rise to a sizable electric potential. When light impinges from the extracellular side, in spite of an effective electron accumulation comparable to the opposite side illumination, we do not observe any polarization due to the effective screening of the reduced oxygen ions accumulating at the surface.

In conclusion, our simulations suggest that i) the almost unipolar *p* transport in P3HT, ii) the presence of oxygen in the retinal environment, iii) the resistivity of the cleft and iv) the illumination side are the main physical ingredients eliciting nanoparticle photostimulation.

Cited references

Bargigia, I., Zucchetti, E., Srimath Kandada, A.R., Moreira, M., Bossio, C., Wong, W., Miranda, P., Decuzzi, P., Soci, C., D'Andrea, C. & Lanzani, G. The photo-physics of polythiophene nanoparticles for biological applications. *ChemBioChem* **19**,1–6 (2018).

Chiaravalli G., Manfredi G., Sacco R. & Lanzani G. Photo-electrochemistry and drift-diffusion simulations in a polythiophene film interfaced with an electrolyte. *ACS Appl. Mater. Interfaces* **13**, 36595–36604 (2021).

Gummel, H.K. A self-consistent iterative scheme for one dimensional steady state transistor calculations. *IEEE Trans. Electron Devices* **11**, 455-465 (1964).

Jerome J. W. *Analysis of Charge Transport.* Springer-Verlag (1996).

Jiang, X., Österbacka, R., Korovyanko, O., An, C., Horovitz, B., Janssen, R. & Vardeny, Z. Spectroscopic studies of photoexcitations in regio-regular and regio-random polythiophene films. *Adv. Funct. Mater.* **12**, 587-597 (2002).

Mori, Y. *A three-dimensional model of cellular electrical activity*. PhD dissertation Department of Mathematics, New York University, New York, NY.

Reinsberg, P.H., Koellisch, A., Bawol, P.P. & Baltruschat, H. K–O_2 electrochemistry: achieving highly reversible peroxide formation. *Phys. Chem. Chem. Phys.* **21**, 4286-4294 (2019).

Sacco, R., Guidoboni, G. & Mauri, A. A Comprehensive Physically Based Approach to Modeling in Bioengineering and Life Sciences (1st ed.). Elsevier Science Publishing Co. (2019).

Scharfetter, D. & Gummel, H.K. Large signal analysis of a silicon read diode oscillator. *IEEE Trans. Electron Devices* 1**6**, 64-77 (1969).

Tullii, G., Desii, A., Bossio, C., Bellani, S., Colombo, M., Martino, N., Antognazza, M.R., Lanzani, G. Bimodal functioning of a mesoporous, light sensitive polymer/electrolyte interface. *Org. Electron.* **46,** 88-98 (2017).

Verticchio Vercellin, A.C., Harris, A., Chiaravalli, G., Sacco, R., Siesky, B., Ciulla, T., Guidoboni, G. Physics-based modeling of Age-related Macular Degeneration - A theoretical approach to quantify retinal and choroidal contributions to macular oxygenation. *Math. Biosc.* **339**,108650 (2021).

Supplementary Figures

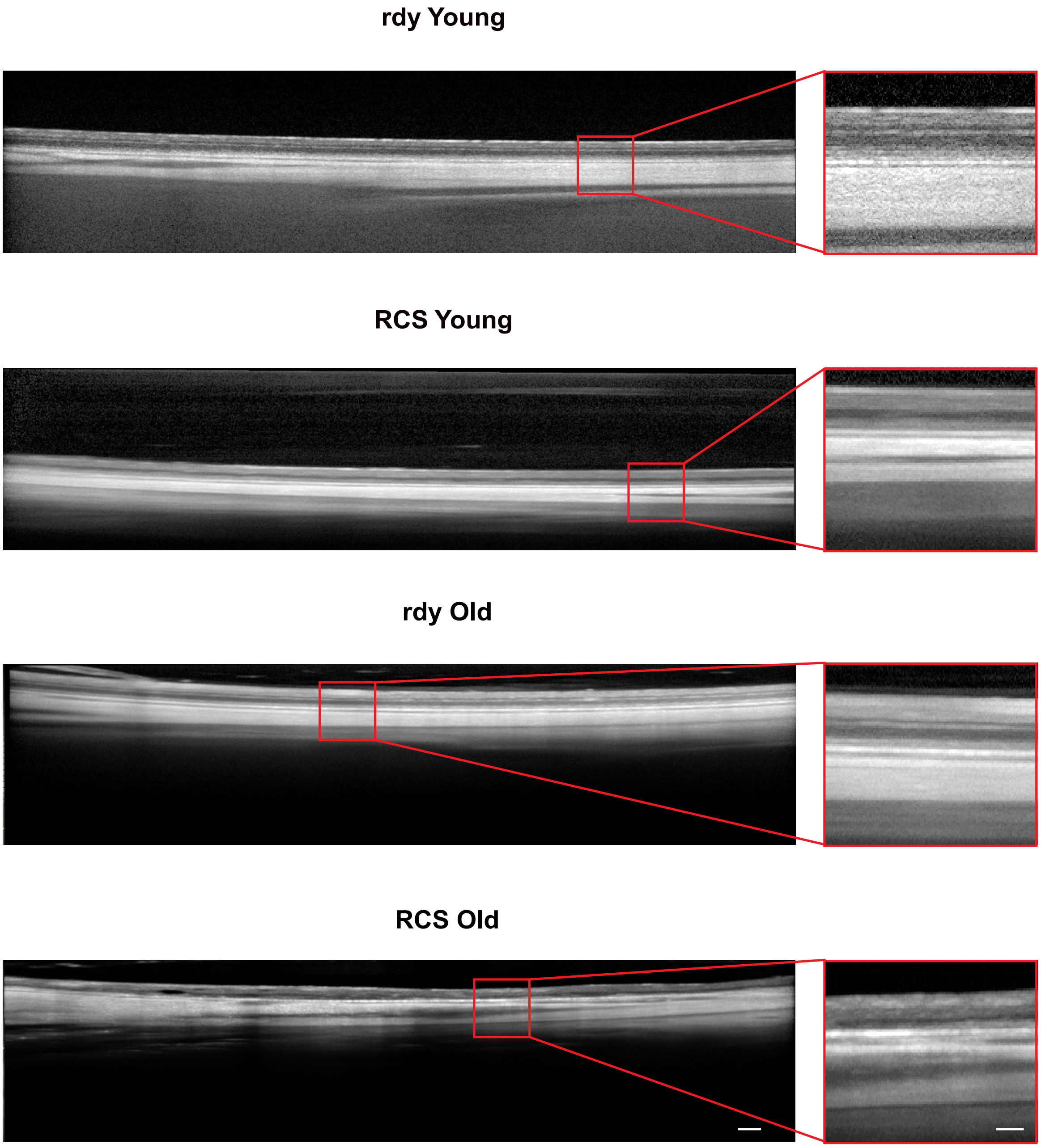

Supplementary Figure 1. Optical coherence tomography (OCT) scans of healthy and dystrophic retinas as a function of age (refers to Figure 1). *Left:* Representative OCT scans of young and aged (2 and 13/15 months of age, respectively) rdy and RCS. *Right:* Higher magnification images of the fields indicated by red squares. Scale bars,100 µm (left), 200 µm (right). Replicates: n = 6 (rdy Young), 5 (RCS Young), 7 (rdy Old), 6 (RCS Old).

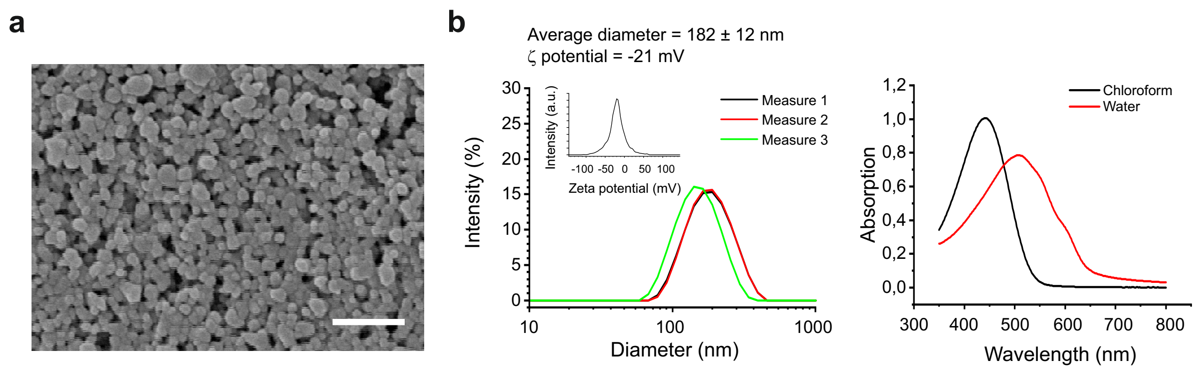

Supplementary Figure 2. Characterization of P3HT-NPs. (a) Scanning electron microscopy (SEM) micrograph of a drop-casted layer of P3HT NPs (scale bar, 2 μm). **(b)** *Left:* Dynamic light scattering (DLS) spectra showing an average diameter of about 182 nm and ζ-potential distribution (inset) with a peak at -21 mV. *Right:* Absorption of a water-suspended P3HT-NPs sample (red trace) and of the same sample of P3HT-NPs re-dissolved in an equivalent amount of chloroform (black trace). From the comparison of chloroform solution absorption with the ones of calibrated solutions, the estimated NP concentration in water was ~ 2,6 mg/ml. To obtain a chloroform solution of P3HT containing the equivalent amount of P3HT contained in water dispersion, 2 ml of water suspension of NPs was poured inside a petri dish. The petri dish was then put on a hot plate at 80 °C and the water was left to evaporate. Once dry, the precipitated nanoparticles were re-dissolved in 2 ml of chloroform. The absorption in the organic solvent is unstructured and blue-shifted with respect to water-suspended NPs due to dissolution of P3HT. The dissolved polymer cannot form crystalline domains, thus reducing the conjugation length and hindering the formation of vibronic replicas. Each DLS measurement was tested 3 times.

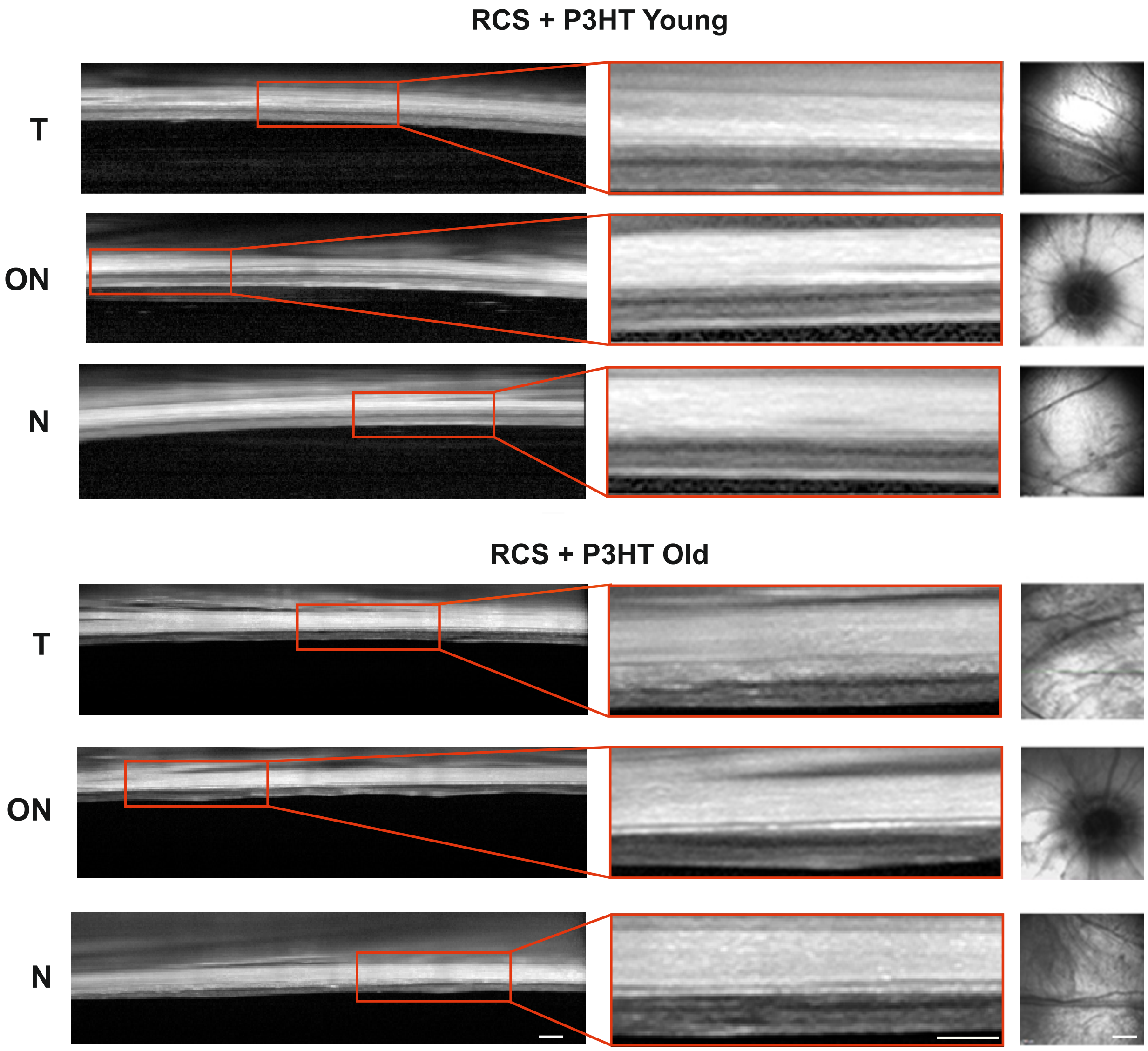

Supplementary Figure 3. Subretinal injection of P3HT-NPs does not affect retinal integrity in young and aged dystrophic RCS rats. Representative images of OCT scans (*left*), higher magnification of the labeled fields (*middle*) and *fundus* (*right*) of young (3-month-old; *upper panels*) and aged (11-month-old; *lower panels*) RCS rats acquired 30 days after the subretinal injection of P3HT-NPs (30 DPI). No retinal damage, signs of inflammation or injection traces were observed in the temporal (T), optic nerve (ON), and nasal (N) areas. Scale bars, 200 μm. Replicates: n = 3 (RCS+P3HT Young), 23 (RCS+P3HT Old).

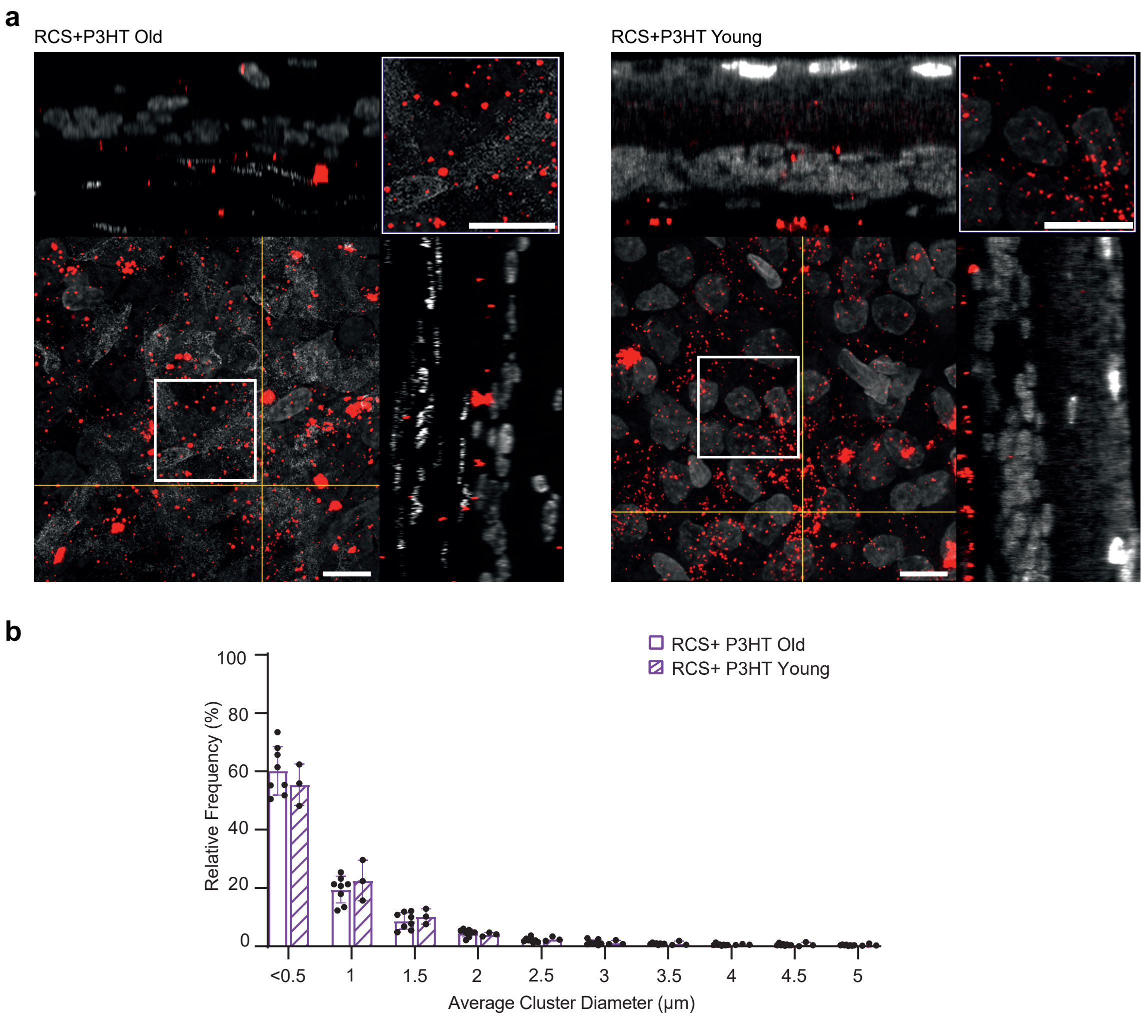

Supplementary Figure 4. P3HT-NPs are highly dispersed in the subretinal space of both young and old RCS rats. (a) Whole-mount retinas from young and aged RCS rats (3- and 13/15-month-old, respectively) subretinally injected with P3HT-NPs are represented by super-resolution confocal z-stack scans in which the P3HT-NP localization (intrinsic fluorescence in red) is clearly visible with respect to retinal cells (nuclear staining with bisbenzimide in white). Each panel represents the z-max with x- and y-orthoslices to highlight the degree of dispersion of the P3HT-NPs. Scale bar, 10 μm. **(b)** Histogram showing the quantification of the average NP cluster diameter in young and aged RCS rats. The estimated diameter of each NP cluster was calculated as the average of the three dimensions of each isolated fluorescence volume's bounding box. About 40-60% of the volumes have diameters below 500 nm in all cases, with an average of almost monodisperse NPs (> 50 %) regardless of age. Data are shown as means ± sem. Sample size: n = 3 (RCS+P3HT Young) and 8 (RCS+P3HT Old).

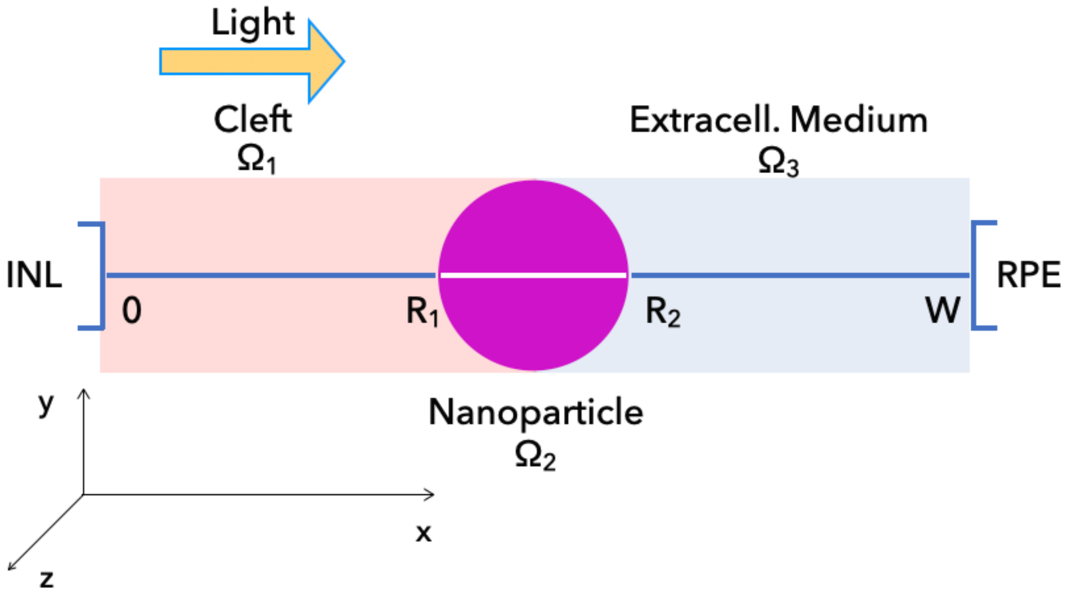

Supplementary Figure 5. 1D computational domain, comprising the nanoparticle Ω_2, the cleft Ω_1 and the extracellular environment Ω_3.

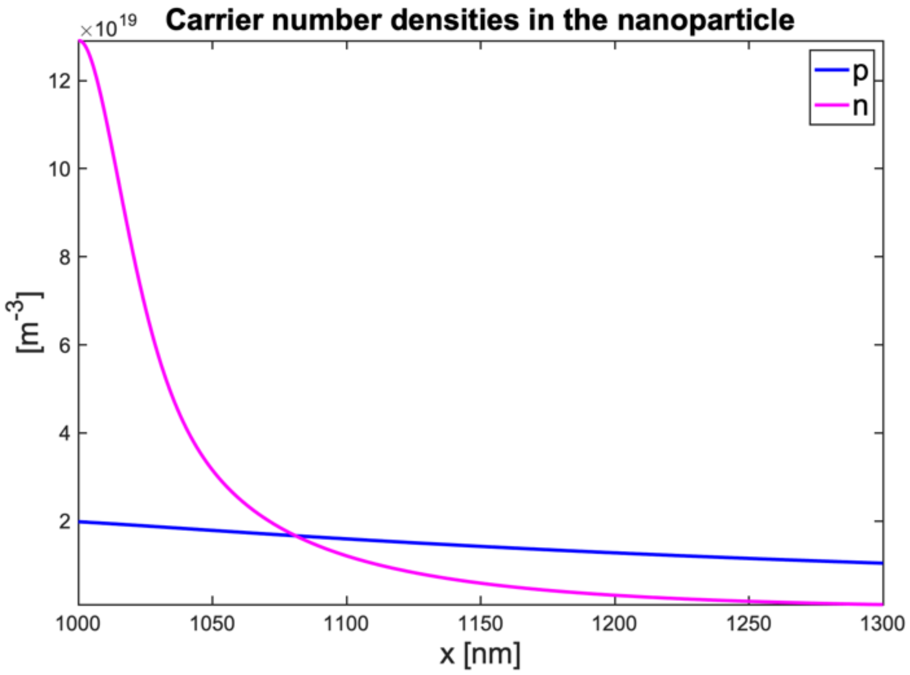

Supplementary Figure 6. Holes and electron number density in the nanoparticle when light is impinging from the cleft side.

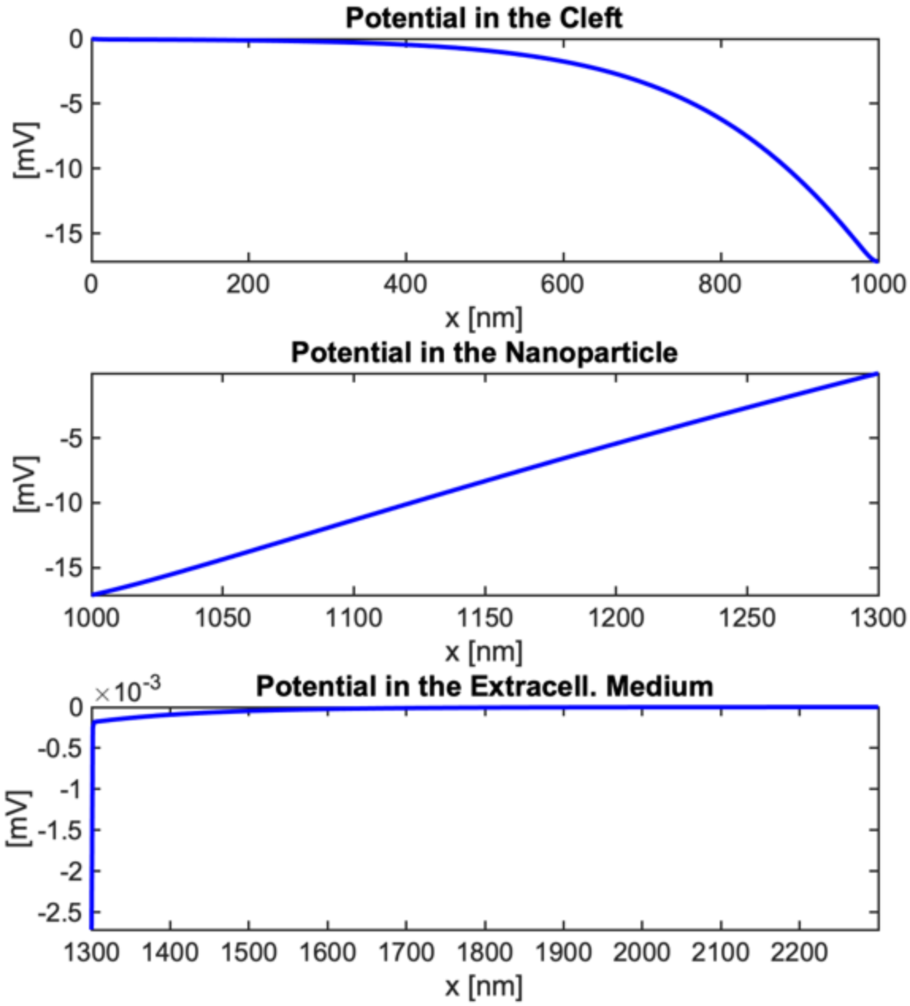

Supplementary Figure 7. Electric potential distributions in the cleft region, in the NP and in the extracellular side.

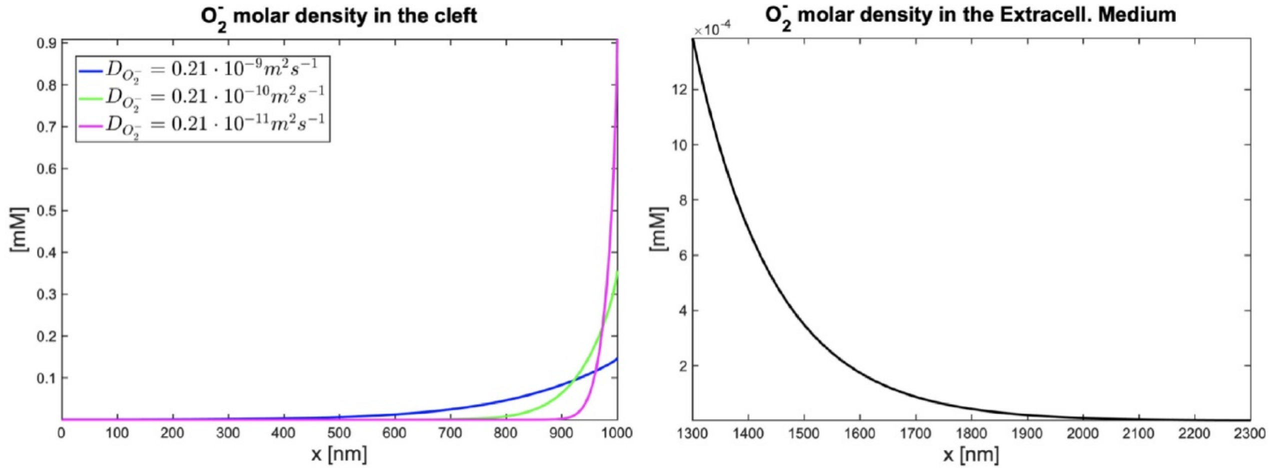

Supplementary Figure 8. O_2^- molar density in the cleft and in the extracellular medium. On the left, the molar concentration is reported varying the value of D_{ox} inside the cleft region. On the right D_{O2-} = 0.21 10^{-9} m^2 s^{-1} as reported in **Supplementary Table 1.**

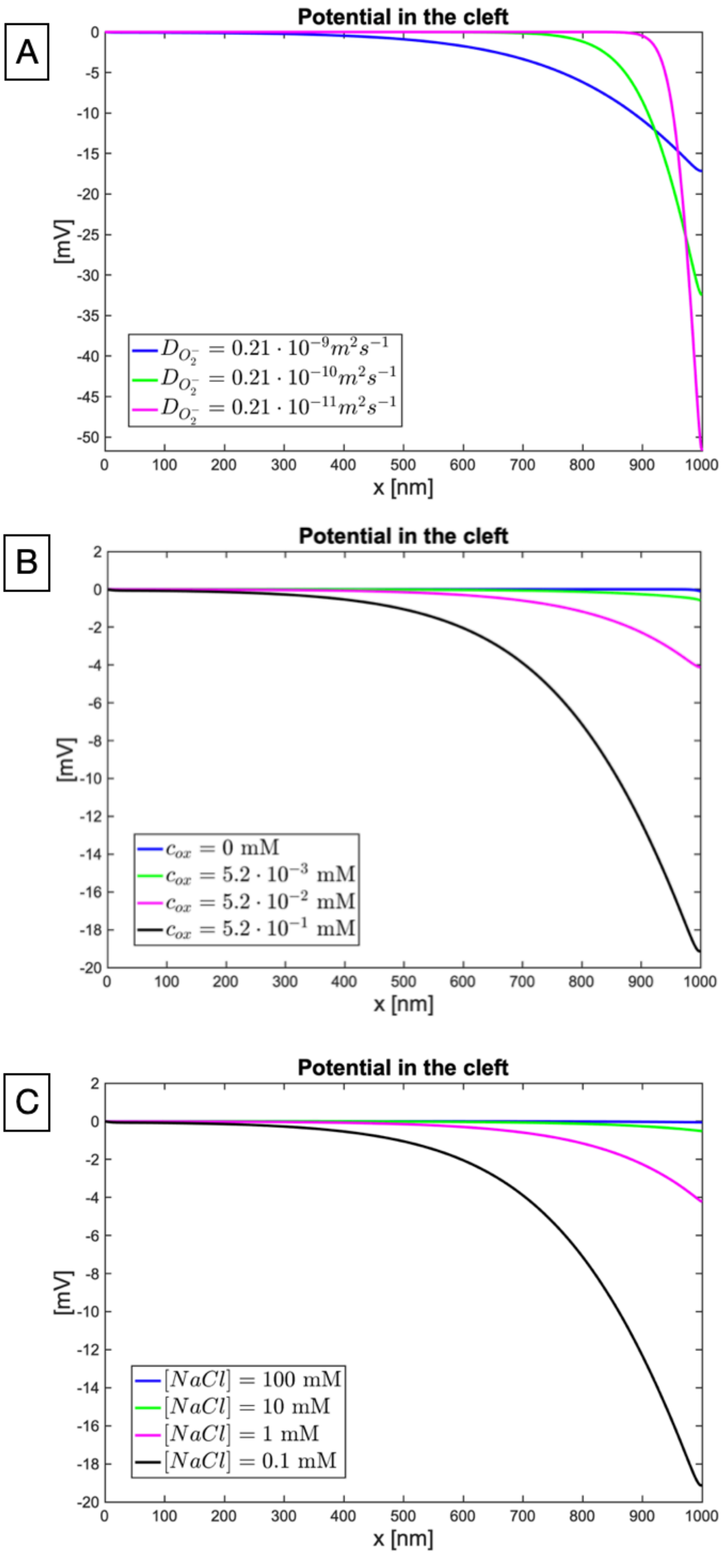

Supplementary Figure 9. Electric potential in the cleft as function of: (**a**) the reduced oxygen diffusion coefficient D_{O2-} ; (**b**) the concentration of molecular oxygen in the cleft; (**C**) the concentration of screening ions, namely Na^+ and Cl^-.

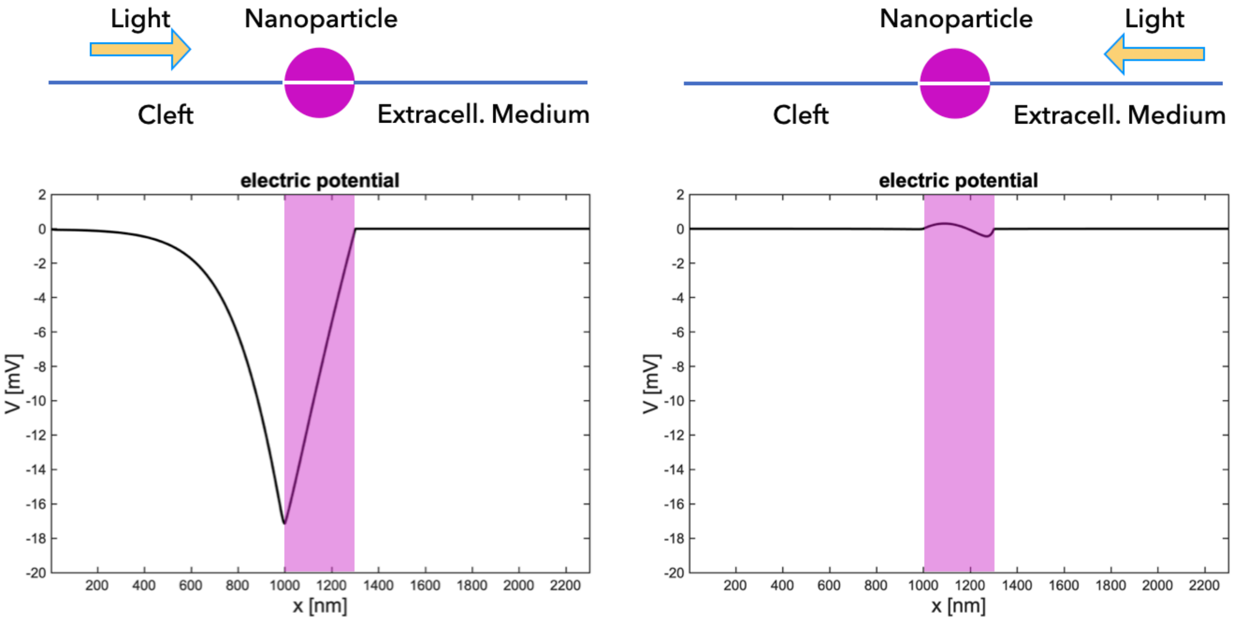

Supplementary Figure 10. Electric potential across the whole domain. *Left panel:* light impinges from the cleft side. *Right Panel:* light impinges from the extracellular medium side. The purple shaded area highlights the nanoparticle domain.

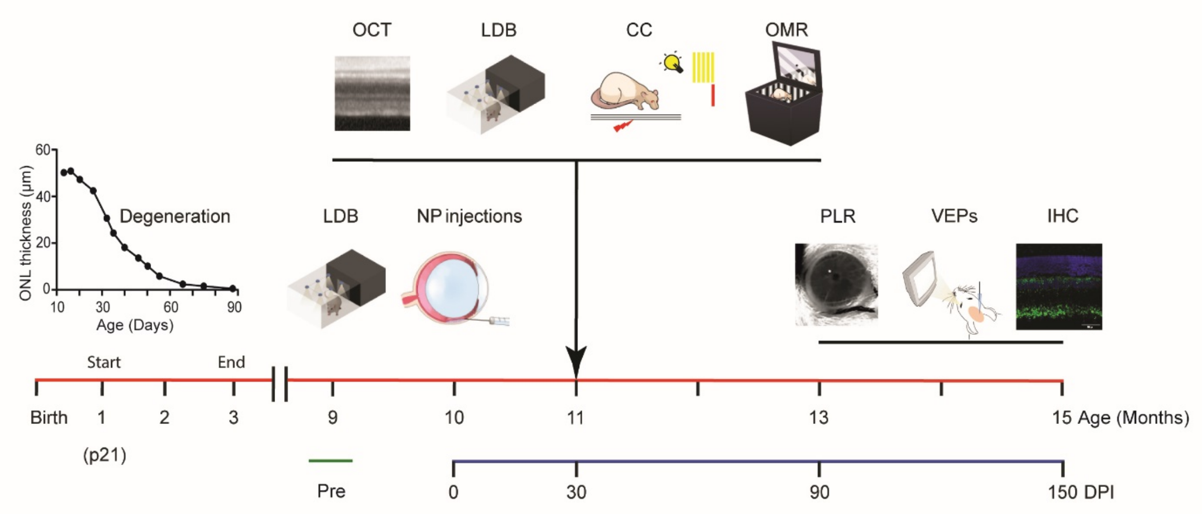

Supplementary Figure 11. Timeline of the experiments for the evaluation of visual performances in aged RCS rat.

The photoreceptors of pink-eyed Royal College of Surgeons (RCS) start degenerating at postnatal day 21 (p21) and, in 2 months, the degeneration is virtually complete, as shown by the progressive thinning of the outer nuclear layer (ONL) the inset graph modified from [31]. At 9 months of age, well beyond the end of the degeneration and one month before the injection of P3HT-NPs (Pre), all animals involved in the experiments underwent a behavioral light-dark box (LDB) test evaluating the light-escape latency. At 10 months, either P3HT-NPs or inert size-matched SiO_2-NPs were subretinally injected in dystrophic RCS rats. At 11 months, 30 days post-injection (30 DPI), rats injected with NPs underwent optical coherence tomography (OCT) scans and behavioral tests including light-dark box (LDB), classical conditioning (CC) and optomotor response (OMR). Between 13 and 15 months (90-150 DPI), pupillary light reflex (PLR) and visually evoked potential (VEP) recordings in response to flash and pattern stimuli were performed. After terminal electrophysiological recordings, eyes were dissected for immunohistochemical (IHC) analysis.

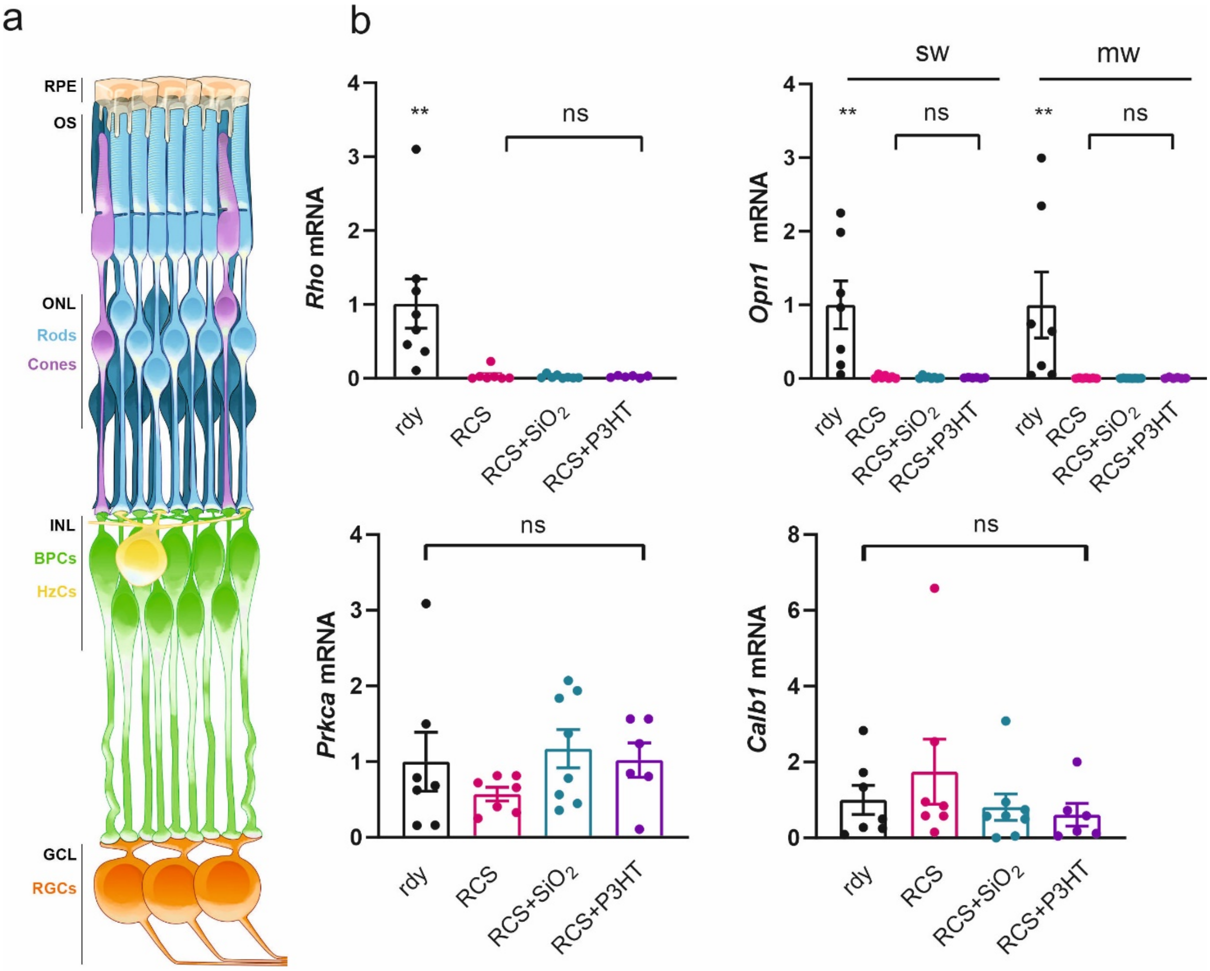

Supplementary Figure 12. Subretinal P3HT-NPs do not affect the expression of neuron-specific mRNAs in aged RCS retinas. (a) Schematic representation of retina layers investigated by qPCR for cell-specific biomarkers. Retinal pigment epithelium (RPE; pink), photoreceptors (rods in light blue and cones in purple), bipolar cells (BPCs; green), horizontal cells (HzCs; yellow) and retinal ganglion cells (RGCs; orange) are shown. OS=outer segment, ONL, outer nuclear layer, INL, inner nuclear layer**,** GCL ganglion cell layer. **(b)** The mRNA levels of the photoreceptor markers Rhodopsin (*Rho*), Opsin-1 short wave-sensitive (*Opn1sw*), Opsin-1 medium wave-sensitive (*Opn1mw*) (*upper panels*) and of the rBPC and HzC markers Protein kinase C-*α* (*Prkca*) and Calbindin1 (*Calb1*) (*lower panels*) were quantified by qRT-PCR in retinal sections dissected at 90-150 DPI from aged (13/15-month-old) non-dystrophic controls (rdy) and dystrophic RCS rats that were non-injected (RCS), injected with P3HT-NPs (RCS+P3HT) or sham-injected with SiO_2-NPs (RCS+SiO_2). *Gapdh* and *Pgk1* were used as reference genes. Graphs show means ± sem with superimposed individual data points. No significant effects of the NP injection on the transcriptional profiles of dystrophic RCS rats were observed, irrespective of the treatment. **p<0.01 rdy *vs* all RCS

groups; ns, not significant; Kruskal-Wallis ANOVA/Dunn's tests. Data are shown as means ± sem with superimposed experimental points. Sample sizes for *Rho* (8,7,8,6); *Opn1sw* (7,7,8,6); *Opn1mw* (7,7,8,6); O*pn4* (7,7,8,6); *Prkca* (7,7,8,6); *Calb1* (7,7,8,6), *Grm6* (7,7,8,4), *Grik1* (7,7,6,5). For exact p values, see Source data file. Source data are provided as a Source data file.

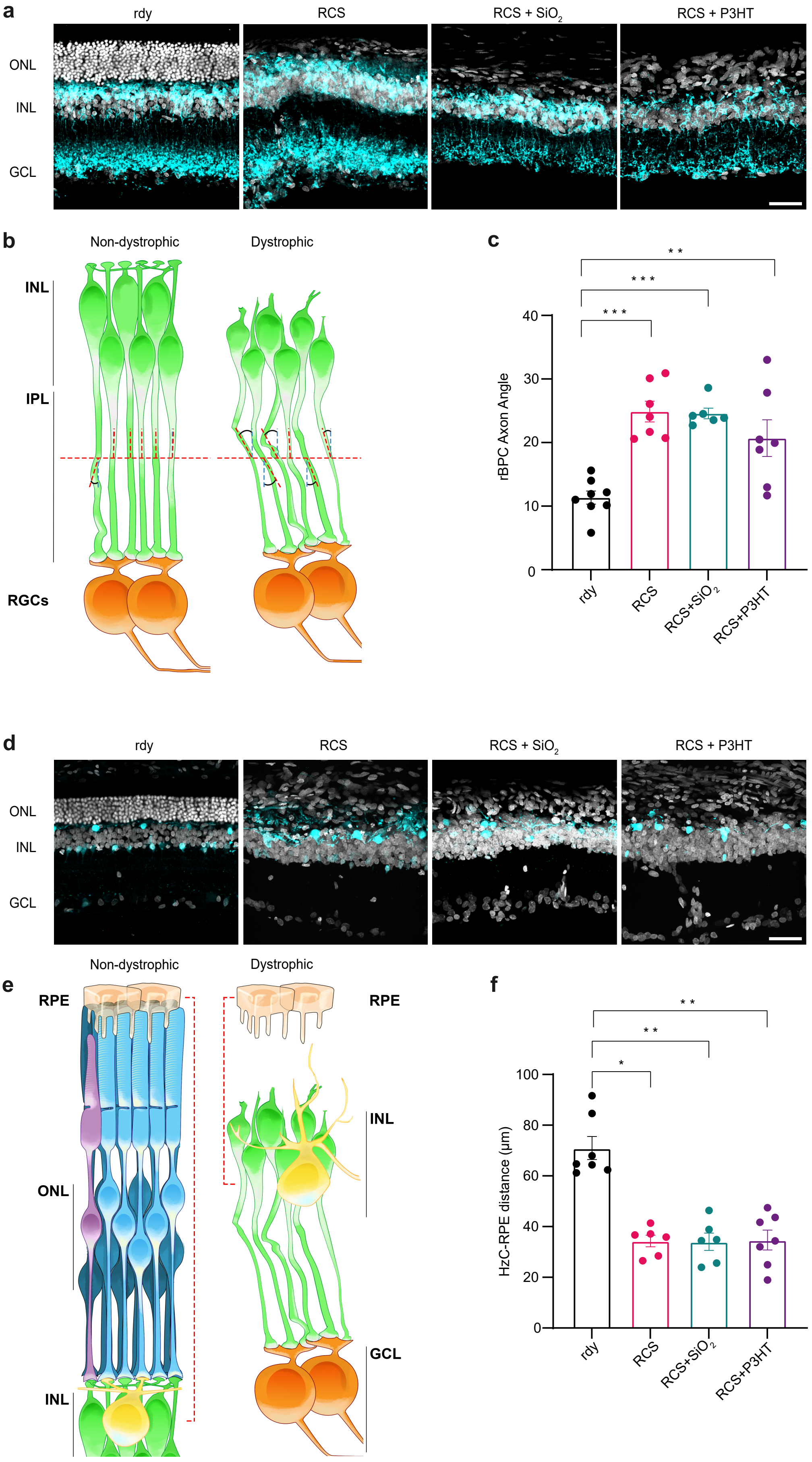
a
rdy
RCS
RCS + SiO2
RCS + P3HT
ONL
INL
GCL
b
Non-dystrophic
Dystrophic
INL
IPL
RGCs
c
rBPC Axon Angle
0
10
20
30
40
rdy
RCS
RCS+SiO2
RCS+P3HT
* * *
* * *
* *
d
rdy
RCS
RCS + SiO2
RCS + P3HT
ONL
INL
GCL
e
Non-dystrophic
Dystrophic
RPE
RPE
ONL
INL
INL
GCL
f
HzC-RPE distance (µm)
0
20
40
60
80
100
rdy
RCS
RCS+SiO2
RCS+P3HT
*
* *
* *

Supplementary Figure 13. Degeneration-induced inner retinal rewiring is not modified by the injection of P3HT-NPs. (**a)** Representative images immunostained for PKCα in injected and non-injected animals at 90-150 DPI (13/15 months of age). **(b)** Schematic representation of the method followed for the evaluation rod BPC (rBPC) axon deviation angle. **(c)** Bar plots (mean ± sem with individual data points) showing the deviation from the physiological right angle of the axon of rBPCs immunostained for PKCα. Sample size: n = 8, 7, 6, 7 for rdy, RCS, RCS+SiO_2 and RCS+P3HT, respectively. **(d)** Representative images immunostained for Calbindin-1 in injected and non-injected animals at 90-150 DPI (13-15 months of age). **(e)** Schematic representation of the method followed for the evaluation of distance of HzC somas from the RPE. **(f)** Bar plots (means ± sem with individual data points) showing the distance of HzCs immunostained for Calbindin-1 from the RPE. one-way ANOVA/Holm-Šídák's tests (c) and Kruskal-Wallis/Dunn (f). *p<0.01, **p<0.05, ***p<0.001; Sample size: n = 7, 6, 6, 7 for rdy, RCS, RCS+SiO_2 and RCS+P3HT, respectively. OS, outer segment; ONL, outer nuclear layer; IPL, inner plexiform layer; INL, inner nuclear layer**;** GCL, ganglion cell layer; rBPC, rod bipolar cell; HzC, horizontal cell; RPE, retinal pigment epithelium. Scale bar, 50 µm. For exact p values, see Source data file. Source data are provided as a Source data file.

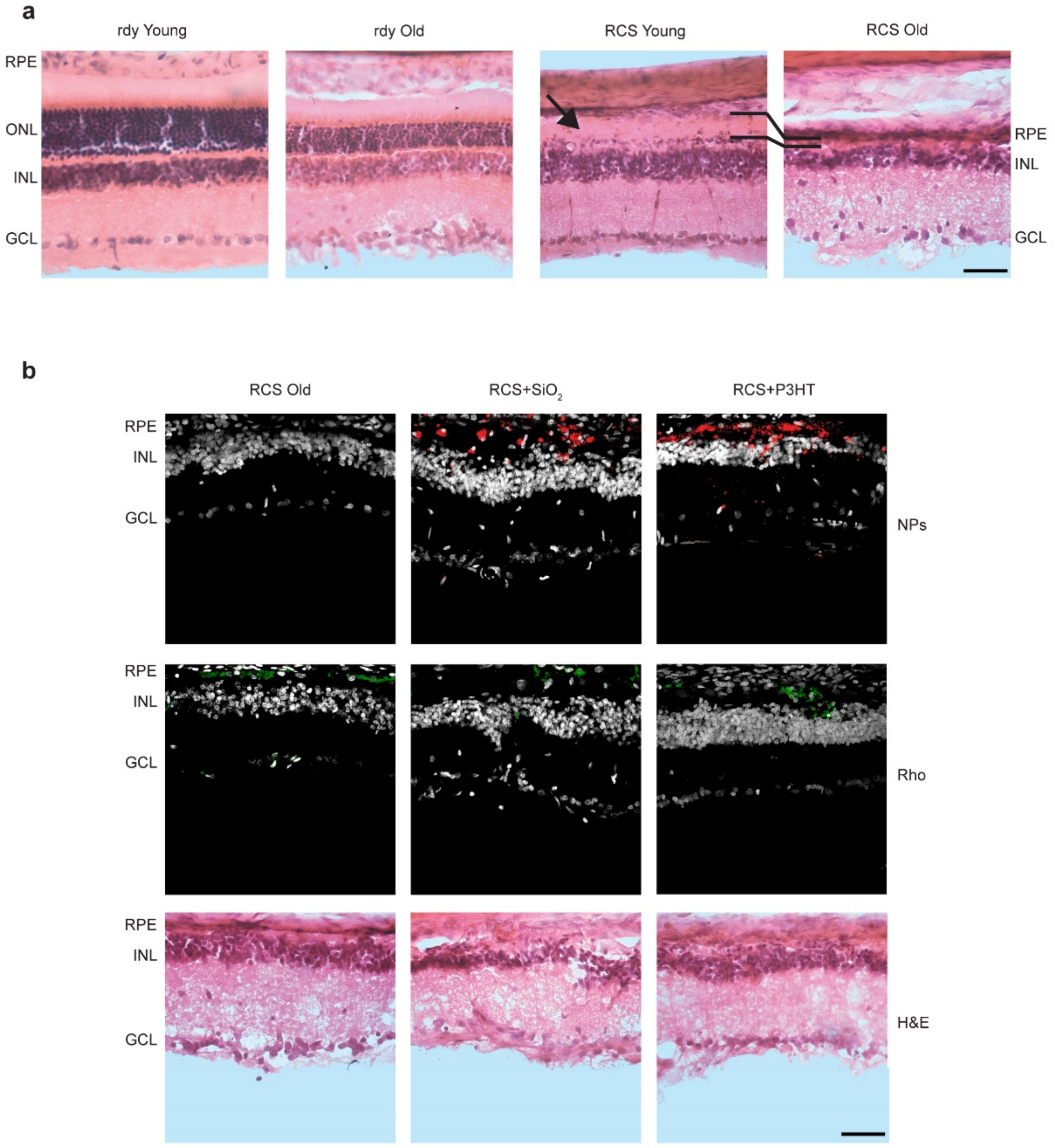

Supplementary Figure 14. Subretinal injection of either P3HT- or SiO_2-NPs does not alter the structure of the outer retina in RCS rats. (a) Representative images of retinal sections from untreated rdy and RCS rats at 3 and 13/15 months of age stained with hematoxylin/eosin. The thick layer of cellular debris (arrow) observed in the outer retina of young animals due the ongoing photoreceptor degeneration is drastically reduced in advanced stage dystrophic retinas of old RCS rats. Sample size: n = 6, 8, 7, 9 for rdy young, rdy Old, RCS young, RCS Old, respectively**. (b)** Representative images of retinal sections from aged (13/15-month-old) RCS rats that were untreated or subretinally injected with either P3HT-NPs or fluorescent SiO_2-NPs. Retinal sections were imaged for NP fluorescence (*upper panels*), immunolabeled for rhodopsin (Rho; *middle panels*) and histologically stained with hematoxylin/eosin (*lower panels*). Sample size: n = 9, 4, 6 for RCS Old, RCS+SiO_2,

RCS+P3HT respectively. Scale bars, 50 μm. RPE, retinal pigment epithelium; INL, inner nuclear layer; GCL ganglion cell layer.

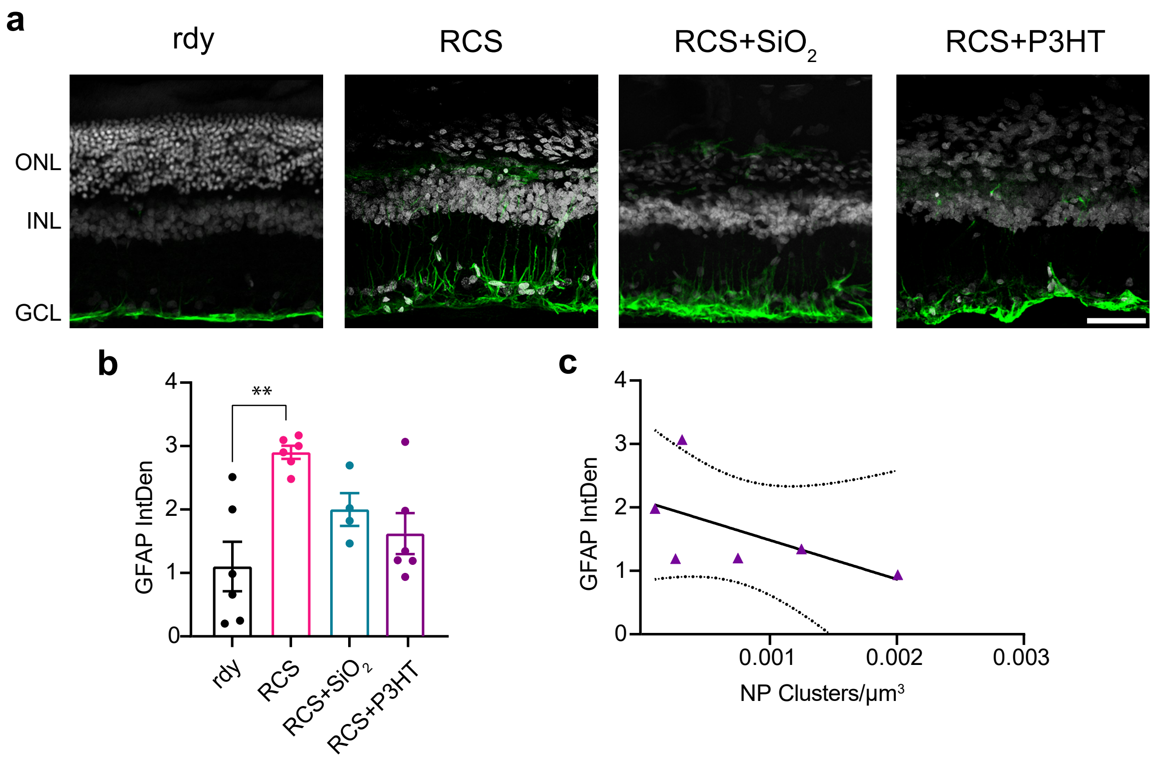

Supplementary Figure 15. P3HT-NPs do not affect retinal astrogliosis due to degeneration in aged RCS rats. Transversal sections of representative retinas dissected at 90-150 DPI from aged (13/15-month-old) non-dystrophic controls (rdy) and dystrophic RCS rats that were non-injected (RCS), injected with P3HT-NPs (RCS+P3HT) or sham-injected with SiO_2-NPs (RCS+SiO_2). **(a)** Representative retinal sections immunolabelled for the astrocyte/Müller cell marker GFAP merged with bisbenzimide nuclear labeling (white). ONL, outer nuclear layer; INL, inner nuclear layer; GCL, ganglion nuclear layer. Scale bar, 50 µm. **(b)** Quantitative analysis of the integrated density of GFAP immunoreactivity (GFAP IntDen). **(c)** Correlation plot between the extent of GFAP expression and the respective density of P3HT-NPs (Pearson's correlation coefficient, 0.57; $p>0.2$; n=6). Dystrophic retinas display higher densities of activated astrocytes compared to rdy rats, due to the degenerative process. The injection of either P3HT- or SiO_2-NPs did not induce any significant increase of retina GFAP expression. In the bar plots, data are expressed as means ± sem with superimposed experimental points. **$p<0.01$, Kruskal Wallis/Dunn's tests; Sample size: n = 6, 6, 4, 6 for rdy, RCS, RCS+SiO_2 and RCS+P3HT, respectively. For exact p values, see Source data file. Source data are provided as a Source data file.

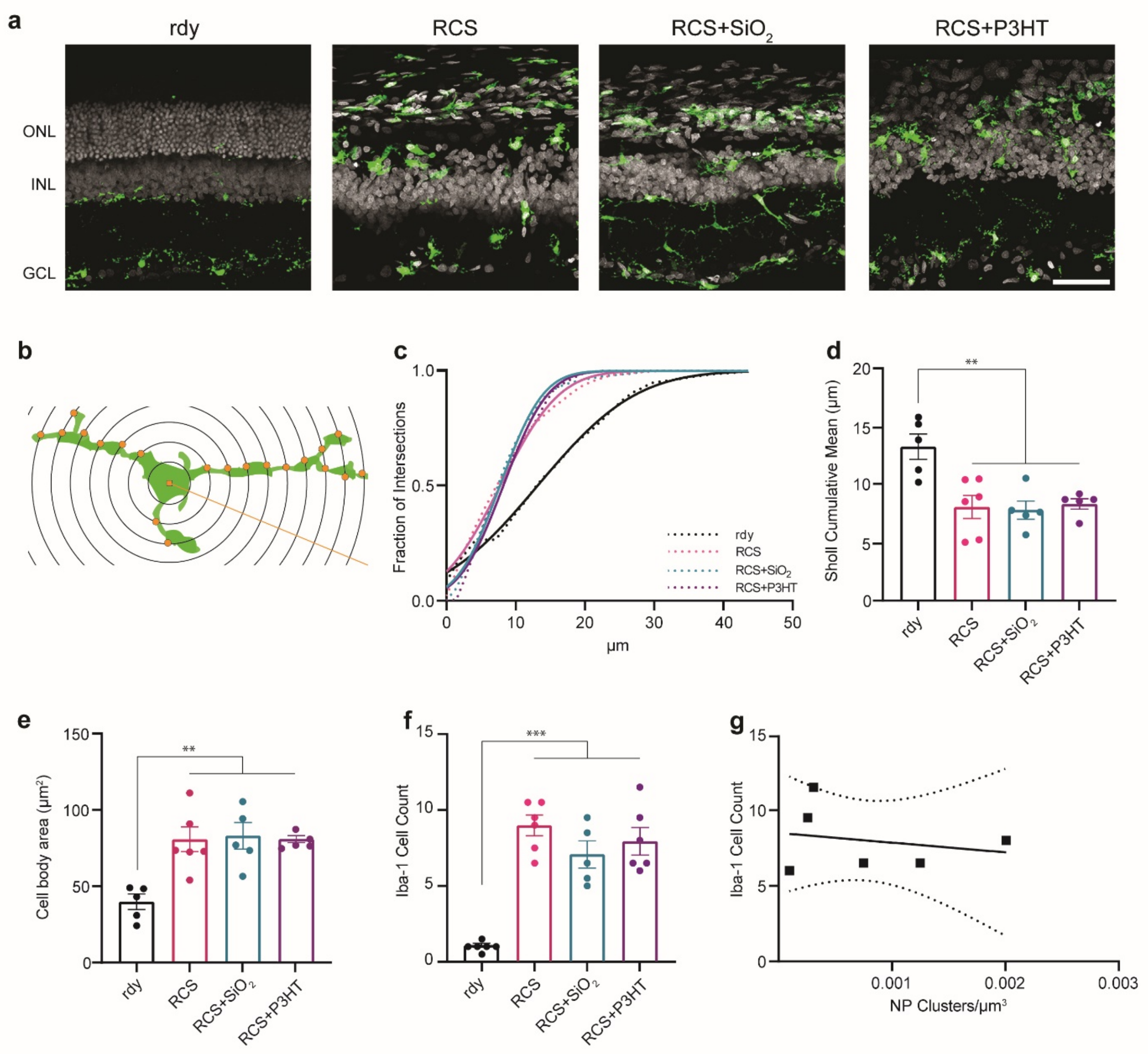

Supplementary Figure 16. P3HT-NPs do not have pro-inflammatory effects in aged RCS retinas. Transversal sections of representative retinas dissected at 90-150 DPI from aged (13/15-month-old) non-dystrophic controls (rdy) and dystrophic RCS rats that were non-injected (RCS), injected with P3HT-NPs (RCS+P3HT) or sham-injected with SiO_2-NPs (RCS+SiO_2). **(a)** Representative retinal sections immunolabelled for the microglial marker Iba-1 merged with bisbenzimide nuclear labeling (white). Microglia in healthy retinas are primarily located in the inner retina, with just a small portion of cells at the border between ONL and INL. Despite photoreceptor degeneration had been completed a long time before the test, old RCS dystrophic rats still show numerous infiltrated microglial cells in the subretinal space. ONL, outer nuclear layer; INL, inner nuclear layer; GCL, ganglion nuclear layer. Scale bar, 50 μm. **(b)** Schematics of the Sholl analysis performed on retinal microglial cells. **(c)** Cumulative sum of intersections as a function of the distance from the soma for each group (dotted lines).

Sample size n = 5, 6, 5, 5 for rdy, RCS, RCS+SiO_2 and RCS+P3HT, respectively. Full lines represent the data fitting with a cumulative Gaussian function. **(d)** Histogram of the Scholl cumulative mean computed by fitting the cumulative curves of intersections from each individual cell. Sample size n = 5, 6, 5, 5 for rdy, RCS, RCS+SiO_2 and RCS+P3HT, respectively. One-way ANOVA/Holm-Šídák's tests. **p<0.01. **(e)** Microglia cell body area. Sample size n = 5, 6, 5, 5 for rdy, RCS, RCS+SiO_2 and RCS+P3HT, respectively. One-way ANOVA/Holm-Šídák's tests. **p<0.01. **(f)** The histogram shows the number of Iba-1 positive cells counted in the ONL. The number of cells is the average from 2 fields per retina. Sample size n = 6, 6, 5, 6 for rdy, RCS, RCS+SiO_2 and RCS+P3HT, respectively. One-way ANOVA/Holm-Šídák's tests. ***p<0.01. **(g)** Correlation plot between the number of Iba1-positive cells and the respective density of P3HT-NPs (Pearson’s correlation coefficient, 0.22; p>0.6; n=6). For exact p values, see Source data file. Source data are provided as a Source data file.

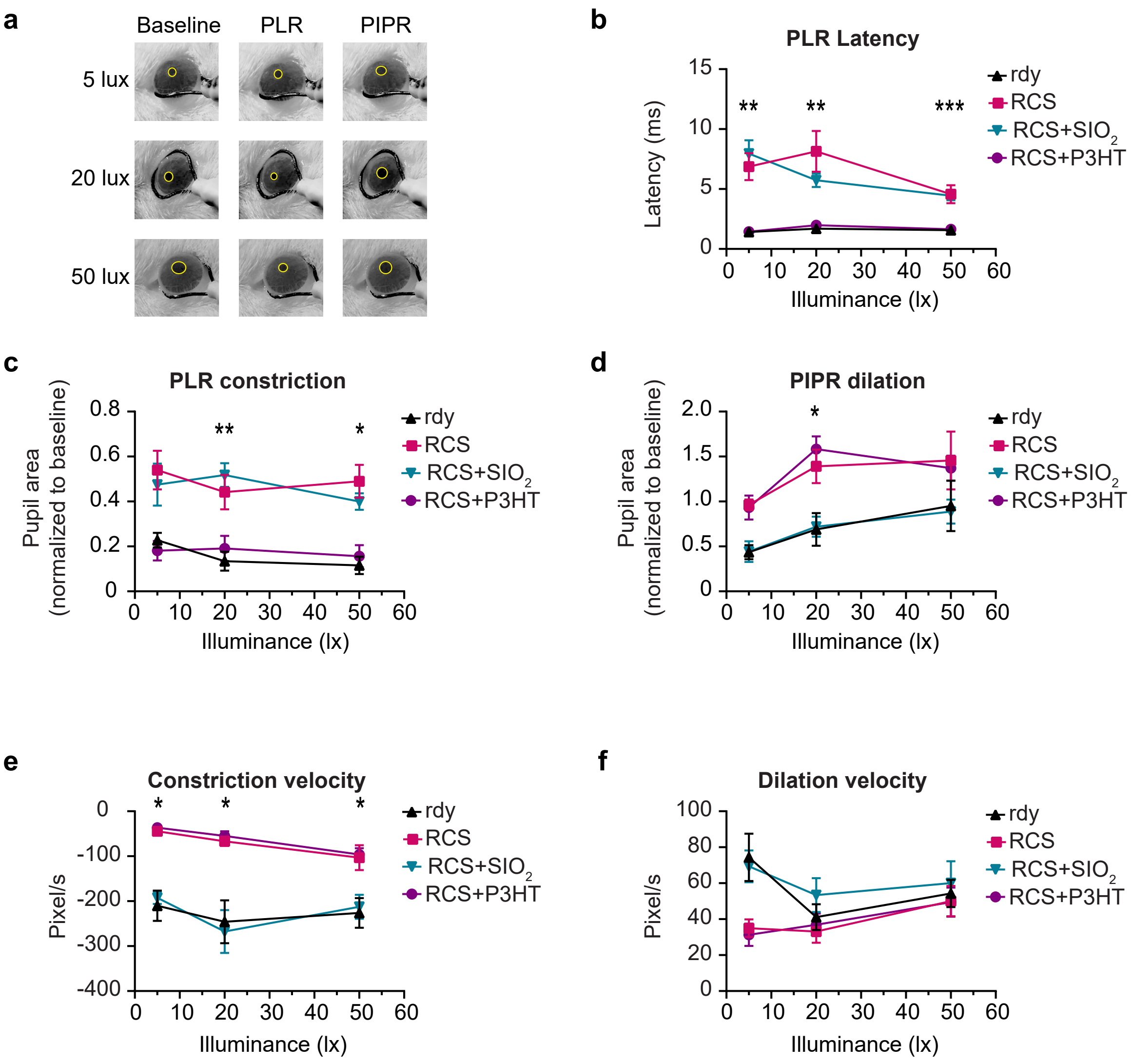

Supplementary Figure 17. Subretinal P3HT-NPs rescue the pupillary light reflex at various luminances. (a) Example of pupil constriction and dilation in response to a prolonged stimulus (20 sec) of green light (530 nm) at three luminances (5, 20, and 50 lux) recorded in aged (13/15-month-old) non-dystrophic controls (rdy) and dystrophic RCS rats that were non-injected (RCS), injected with P3HT-NPs (RCS+P3HT) or sham-injected with SiO_2-NPs (RCS+SiO_2). The video was acquired under infrared illumination to track and measure the pupil area variations during the experiment. Low-quality videos have been discarded; consequently, animal numbers within the same group may slightly differ among different luminances. **(b)** *PLR latency* (msec) from the light switch to the start of pupillary constriction. The PLR latency of rdy and RCS+P3HT groups is significantly shorter than that recorded in RCS and RCS+SiO_2 groups at all tested luminances. **(c)** *Pupillary constriction* normalized to baseline. The extent of pupillary constriction of RCS+P3HT injected rats is comparable to the rdy group and significantly different from the RCS and RCS+SiO_2 groups at all tested

luminances. **(d)** *PIPR dilation* normalized to baseline. RCS+P3HT and rdy animals show significantly smaller dilation after illumination than RCS and RCS+SiO_2 groups at 5 and 20 lux, but not at 50 lux. **(e)** *Constriction velocity.* RCS+P3HT and rdy animals show a significantly faster velocity of constriction compared to RCS and RCS+SiO_2. **(f)** *Dilation velocity*. RCS+P3HT and rdy groups dilate faster than RCS and RCS+SiO_2 at 5 lux, while no significant differences were observed at 20 and 50 lux. In the plots, means ± sem are shown. Asterisks refer to the comparisons RCS+P3HT *versus* the RCS+SiO_2 sham-group. *p<0.05, **p<0.01, ***p<0.01; Mixed-effects model with the Geisser-Greenhouse correction followed by Šídák's multiple comparisons test. For sample size (n) of each experimental group and exact p values, see Source data file. Source data are provided as a Source data file.

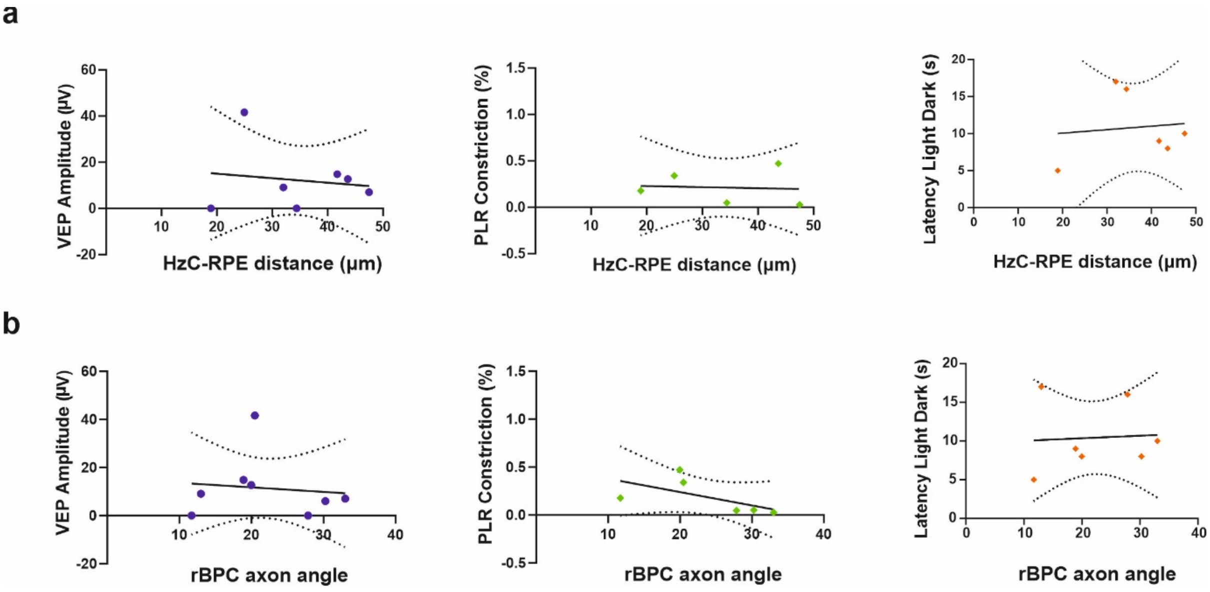

Supplementary Figure 18. The extent of inner retina rewiring does not affect the P3HT-NP-dependent visual rescue. Linear regression analysis of the correlation between the level of visual rescue and the degree of inner retina remodeling. VEP amplitude (left), pupillary constriction (PLR; *middle*) and behavioral escape latency to light (*right*) are plotted, in the same individuals of P3HT-NP injected aged RCS rats, against the distance of horizontal cells (HzC) from RPE **(a)** and the rod bipolar cell (rBPC) axon angle **(b)**. No correlations are observed between visual performances and the severity of inner retinal reorganization. HzC-RPE distance: VEP amplitude: p=0.76, r=0.14, n=7; Pupillary Constriction: p=0.9, r=0.07, n=5; Light Dark Latency: p=0.84, r=0.10, n=6. rBPC axon angle: VEP amplitude: p=0.8, r=-0.11, n= 8; Pupillary Constriction: p=0.20, r=-0.6, n=6; Light Dark Latency: p=0.89, r=0.06, n=7. For exact p values, see Source data file. Source data are provided as a Source data file.

Supplementary Table 1. Parameter values of the mathematical model.

Parameters	Units	Value	Source
$\boldsymbol{D_{Na^+}}$	$m^2\,s^{-1}$	$\mathbf{1.33 \cdot 10^{-9}}$	(Mori, 2006)
$\boldsymbol{D_{Cl^-}}$	$m^2\,s^{-1}$	$\mathbf{2.03 \cdot 10^{-9}}$	(Mori, 2006)
$\boldsymbol{D_{O_2^-}}$	$m^2\,s^{-1}$	$\mathbf{0.21 \cdot 10^{-9}}$	(Reinsberg et al., 2019)
$\boldsymbol{\tau_{ox}}$	s	$\mathbf{1 \cdot 10^{-4}}$	-
$\boldsymbol{I_0}$	$mW\,cm^{-2}$	$\mathbf{10}$	-
$\boldsymbol{k_t}$	$m^4\,s^{-1}$	$\mathbf{5 \cdot 10^{-29}}$	Adapted from (Chiaravalli et al., 2021)
$\boldsymbol{k_p}$	$m^4\,s^{-1}$	$\mathbf{5 \cdot 10^{-29}}$	-
$\boldsymbol{c_{ox}}$	$mol\,m^{-3}$	$\mathbf{0.5}$	Calculated from (Verticchio Vercellin et al., 2021)

Supplementary Table 2. Primary antibody dilutions for the immunolabeling of retinal sections.

Primary antibody	Localization	Supplier	Cat. No.	Clone	Host	Type	Dilution
**Anti-PKCα**	Rod Bipolar Cells	Santa Cruz	sc-8393	H-7	Mouse	Monoclonal	1:300
**Anti-Calbindin1 D28k**	Horizontal Cells	Swant	300		Mouse	Monoclonal	1:250
**Anti-GFAP**	Müller Cells	Sigma	G3893	G-A-5	Mouse	Monoclonal	1:250
**Anti-Iba1**	Microglia	Wako	019-19741		Rabbit	Polyclonal	1:500
**Anti-Rhodopsin**	Rod outer segments	Merck	MABN15	4D2	Mouse	Monoclonal	1:500
**Anti-Cone arrestin**	Cone photoreceptors	Merck	AB15282		Rabbit	Polyclonal	1:250

Supplementary Table 3. List of the primers used in the qRT-PCR analysis.

Gene Symbol	Description	Forward sequence	Reverse sequence
**Calb1**	calbindin 1	GATCACAGCCTCACAGTT	CTCCAATCCAGCCTTCTT
**Gapdh**	glyceraldehyde-3-phosphate dehydrogenase	CCATTCTTCCACCTTTGA	CTGTAGCCATATTCATTGTC
**Opn1mw**	opsin 1, medium wave sensitive	CCACTATCTACAATCCCATT	CTATCATCAACTTTCTTTCCAA
**Opn1sw**	opsin 1, short wave sensitive	ACGTTCTGGTCAATGTAT	CCAAAGAGGAAGTATCCAT
**Pgk1**	phosphoglycerate kinase 1	ATGCAAAGACTGGCCAAGCTAC	AGCCACAGCCTCAGCATATTTC
**Prkca**	protein kinase C alpha	CTGGGAGAAGTTGGAGAA	CGTGTGAAGAACTTGTCAA
**Rho**	rhodopsin	CCCTCAGTGTTCTTTTCT	GATGGATGTCCTTTGTCA
