# Supplementary material for: Light-induced charge generation in polymeric nanoparticles restores vision in advanced-stage retinitis pigmentosa rats
Source: Nat Commun. 2022 Jun 27;13:3677. doi: 10.1038/s41467-022-31368-3 (PMC9237035; doi:10.1038/s41467-022-31368-3)
Supplement: Supplementary file 3 — Description of additional Supplementary File [file 41467_2022_31368_MOESM3_ESM.pdf]

<u>Descriptions of additional supplementary data files</u>

Supplementary Video 1. : Pupillary reflexes in bilaterally injected and dark-adapted 11-months-old animals at 30 DPI evoked by a light stimulus of 20 lux. Movies are image sequences obtained with a Hamamatsu camera at 5 Hz frame rate. Representative examples of the four experimental groups (RCS-rdy; RCS; RCS+P3HT-NPs; RCS+SiO2-NPs) are shown.

Supplementary Video 2: Escape latency in the light dark-box test in 11-months-old animals at 30 DPI. Representative examples of the 5-lux light-evoked escape behavior for the four age-matched experimental groups (rdy, RCS, RCS+SiO2, RCS+P3HT) are shown.

Supplementary Software: Source MATLAB code for morphological analysis and modeling simulations
