# Supplementary material for: Light-induced charge generation in polymeric nanoparticles restores vision in advanced-stage retinitis pigmentosa rats
Source: Nat Commun. 2022 Jun 27;13:3677. doi: 10.1038/s41467-022-31368-3 (PMC9237035; doi:10.1038/s41467-022-31368-3)
Supplement: Supplementary file 7 — Reporting Summary [file 41467_2022_31368_MOESM7_ESM.pdf]

Corresponding author(s): Fabio Benfenati, Guglielmo Lanzani

Last updated by author(s): May 16, 2022

Reporting Summary

Nature Portfolio wishes to improve the reproducibility of the work that we publish. This form provides structure for consistency and transparency in reporting. For further information on Nature Portfolio policies, see our Editorial Policies and the Editorial Policy Checklist.

Statistics

For all statistical analyses, confirm that the following items are present in the figure legend, table legend, main text, or Methods section.

n/a	Confirmed	
☐	☒	The exact sample size (*n*) for each experimental group/condition, given as a discrete number and unit of measurement
☐	☒	A statement on whether measurements were taken from distinct samples or whether the same sample was measured repeatedly
☐	☒	The statistical test(s) used AND whether they are one- or two-sided<br>*Only common tests should be described solely by name; describe more complex techniques in the Methods section.*
☐	☒	A description of all covariates tested
☐	☒	A description of any assumptions or corrections, such as tests of normality and adjustment for multiple comparisons
☐	☒	A full description of the statistical parameters including central tendency (e.g. means) or other basic estimates (e.g. regression coefficient) AND variation (e.g. standard deviation) or associated estimates of uncertainty (e.g. confidence intervals)
☐	☒	For null hypothesis testing, the test statistic (e.g. *F*, *t*, *r*) with confidence intervals, effect sizes, degrees of freedom and *P* value noted<br>*Give P values as exact values whenever suitable.*
☒	☐	For Bayesian analysis, information on the choice of priors and Markov chain Monte Carlo settings
☒	☐	For hierarchical and complex designs, identification of the appropriate level for tests and full reporting of outcomes
☐	☒	Estimates of effect sizes (e.g. Cohen's *d*, Pearson's *r*), indicating how they were calculated

Our web collection on statistics for biologists contains articles on many of the points above.

Software and code

Policy information about availability of computer code

Data collection	Electrophysiological data were collected with a custom matlab script coupled with a NIDAQ board. No commercial software were used to collect the other experimental data.
Data analysis	Eye Explorer 3.2.1.0 (Spectralis), Prism 6.07 & 9 (GraphPad), OriginPro 2020 SR1 (OriginLab), ImageJ 1.53c (NIH), Matlab R2019a (MathWorks)

For manuscripts utilizing custom algorithms or software that are central to the research but not yet described in published literature, software must be made available to editors and reviewers. We strongly encourage code deposition in a community repository (e.g. GitHub). See the Nature Portfolio guidelines for submitting code & software for further information.

Data

Policy information about availability of data

All manuscripts must include a data availability statement. This statement should provide the following information, where applicable:

- Accession codes, unique identifiers, or web links for publicly available datasets
- A description of any restrictions on data availability
- For clinical datasets or third party data, please ensure that the statement adheres to our policy

Source data for all figures is provided in a supplementary excel file.

Field-specific reporting

Please select the one below that is the best fit for your research. If you are not sure, read the appropriate sections before making your selection.

☒ Life sciences ☐ Behavioural & social sciences ☐ Ecological, evolutionary & environmental sciences

For a reference copy of the document with all sections, see nature.com/documents/nr-reporting-summary-flat.pdf

Life sciences study design

All studies must disclose on these points even when the disclosure is negative.

Sample size	Sample Size has been calculated with GPower 3.2 software by assuming a size effect based on previous experiments conducted in our laboratory and imposing at least a power of 80%.
Data exclusions	Treated animals (subretinal injections) that displayed retinal detachment or breakage (OCT analysis) or cataract were preliminarily excluded from the analyses. Statistical analysis was also applied to remove outliers following the ROUT algorithm at 1%.
Replication	There were no findings that were not replicated across distinct experimental sessions. Reproducibility ofthe data was assessed by statistically evaluating the intraexperimental and interexerimental variability. Replicate numbers are indicated in corresponding figure legends and Supplementary Table 3. Unless otherwise stated, all representative images were taken from quantified datasets.
Randomization	Animals were randomly selected among available age-matched litters and placed into experimental groups while maintaining a balance between females and males.
Blinding	Experiments were carried out blinded to the experimenter.

Reporting for specific materials, systems and methods

We require information from authors about some types of materials, experimental systems and methods used in many studies. Here, indicate whether each material, system or method listed is relevant to your study. If you are not sure if a list item applies to your research, read the appropriate section before selecting a response.

Materials & experimental systems

n/a	Involved in the study	
☐	☒	Antibodies
☒	☐	Eukaryotic cell lines
☒	☐	Palaeontology and archaeology
☐	☒	Animals and other organisms
☒	☐	Human research participants
☒	☐	Clinical data
☒	☐	Dual use research of concern

Methods

n/a	Involved in the study	
☒	☐	ChIP-seq
☒	☐	Flow cytometry
☒	☐	MRI-based neuroimaging

Antibodies

Antibodies used	Primary antibodies: Mouse monoclonal anti-PKCα H-7 (1:300, Santa Cruz sc-8393); Mouse monoclonal anti-Calbindin1 D-28k (1:250, Swant 300); Mouse monoclonal anti-GFAP G-A-5 (1:250, Sigma G3893); Rabbit polyclonal anti-Iba1 (1:500, Wako 019-19741); Mouse monoclonal anti-Rhodopsin 4D2 (1:500, Merck MABN15); Rabbit polyclonal anti-Cone arrestin (1:250, Merck AB15282); Secondary antibodies: Alexa Fluor 488-conjugated anti-rabbit and anti-mouse hosted in goat. (Molecular Probes, Invitrogen)
Validation	anti- GFAP, Iba1, Cone Arrestin and Rhodopsin staining replicated dilutions as previously published in Maya-Vetencourt et al, Nat Nanotechnol 2020. Anti- PKCα and Calbindin1 primary antibodies were tested at a range of dilutions (1:1000 to 1:200) on healthy RCS-rdy retina slices for optimal immunofluorescent marking.

Animals and other organisms

Policy information about studies involving animals; ARRIVE guidelines recommended for reporting animal research

Laboratory animals	Royal College of Surgeons and healthy congenic ECS-rdy rats of both sexes and ages ranging from 3 to 15 months (see details in online methods)
Wild animals	No wild animals were used in this study.
Field-collected samples	No field-collected samples were involved in this study.

Ethics oversight	All animal manipulations and procedures were performed in accordance with the guidelines established by the European Community Council (Directive 2012/63/EU of 22 Septempber 2010) and were approved by the italian Ministry of Health (Authorization #357/2019-PR)

Note that full information on the approval of the study protocol must also be provided in the manuscript.
